# Inhibition of Galectin-1 and Androgen Receptor Axis Enhances Enzalutamide Treatment in Enzalutamide Resistant Prostate Cancer

**DOI:** 10.3390/cancers17030351

**Published:** 2025-01-22

**Authors:** Hsiao-Chi Wang, Allen C. Gao, Roger Xia, Chun-Te Wu, Ssu-Wei Hsu, Ching-Hsien Chen, Tsung-Chieh Shih

**Affiliations:** 1 Department of Research and Development, Kibio Inc., Houston, TX 77021, USA; 2 Department of Urologic Surgery, University of California at Davis, Davis, CA 95718, USA; 3 Department of Biomedical Data Science, Stanford University, Stanford, CA 94305, USA; 4 Department of Urology, Chang Gung Memorial Hospital, Linko, Taoyuan 333423, Taiwan; 5 Divisions of Nephrology and Pulmonary, Critical Care and Sleep Medicine, Department of Internal Medicine, University of California at Davis, Davis, CA 95616, USA; 6 Comprehensive Cancer Center, University of California at Davis, Davis, CA 95616, USA; 7 Department of Translational Molecular Pathology, The University of Texas MD Anderson Cancer Center, 2450 Holcombe Boulevard, Houston, TX 77021, USA

**Keywords:** prostate cancer, Galectin-1, LLS30

## Abstract

Background/Objective: Prostate cancer (PCa) remains a prevalent and deadly disease, particularly in its advanced stages. Despite various available treatments, resistance to drugs like enzalutamide continues to present significant challenges. This study aimed to investigate the role of Galectin-1 (Gal-1) in enzalutamide-resistant PCa and assess its potential as a therapeutic target to overcome resistance. Methods: The study utilized specific siRNA-mediated knockdown of Gal-1 in enzalutamide-resistant PCa cells to evaluate its effects on cell proliferation and response to enzalutamide treatment. An orthotopic mouse model was employed to examine the in vivo impact of Gal-1 knockdown. Pharmacological targeting of Gal-1 was conducted using LLS30, and its effects were assessed both in vitro and in vivo. RNA sequencing (RNA-seq) analysis was performed to explore the molecular mechanisms underlying the observed effects. Results: The findings demonstrated significant upregulation of Gal-1 in enzalutamide-resistant PCa cells. Gal-1 knockdown inhibited cell proliferation and resensitized resistant cells to enzalutamide treatment in the orthotopic mouse model. Elevated levels of androgen receptor full-length and AR-V7 are key mechanisms under-lying resistance to enzalutamide in PCa. Gal-1 knockdown suppressed AR and AR-V7 expression and their transcriptional activity. Treatment with LLS30 significantly suppressed the growth of enzalutamide-resistant PCa cells and exhibited synergistic effects when combined with enzalutamide. Notably, this combination therapy significantly inhibited the growth of enzalutamide-resistant xenografts in vivo. RNA-seq analysis revealed that LLS30 modulates AR and AR-V7 signaling through the inhibition of associated target genes. Conclusion: These findings highlight Gal-1 as a promising therapeutic target for overcoming enzalutamide resistance in PCa. Targeting the Gal-1/AR/AR-V7 axis with LLS30 presents a novel strategy to enhance enzalutamide efficacy and address drug resistance in advanced PCa.

## Simple Summary

Prostate cancer is a leading cause of cancer death in men, and many patients develop resistance to current treatments like enzalutamide. This study explores the role of Galectin-1, a protein linked to cancer progression, in resistance to enzalutamide. We found that Galectin-1 is upregulated in enzalutamide-resistant prostate cancer cells and promotes resistance by regulating androgen receptor expression. Inhibiting Galectin-1 improved the effectiveness of enzalutamide in both lab and animal models. These findings suggest that targeting Galectin-1 could enhance treatment outcomes for patients with resistant prostate cancer.

## 1. Introduction

Prostate cancer (PCa) is the most prevalent cancer and the second leading cause of cancer death among men. Androgen deprivation therapy is typically the first-line treatment [1]. However, metastatic castration-resistant prostate cancer (mCRPC) remains a significant challenge, with most patients experiencing disease progression within 16 to 18 months due to persistent activation of the androgen receptor (AR) signaling pathway, a key driver of CRPC progression [2,3]. Enzalutamide, an AR inhibitor, is used to treat CRPC by binding to the AR’s ligand-binding domain and inhibiting its activity [4]. Despite this, about a quarter of patients do not respond to initial treatment, and resistance develops within 24 months for many who do initially respond [5,6].

Changes in mRNA splicing, like the development of the androgen receptor variant 7 (AR-V7) that lacks the ligand-binding domain, play a key role in resistance to enzalutamide in CRPC [7,8,9]. This resistance is driven by transcriptional regulation, alternative splicing, and epigenetic modifications. Post-transcriptional modifications, including microRNAs and RNA-binding proteins, along with signaling pathway interactions, also contribute to AR-V7 expression [10,11,12,13]. For instance, proteins like Sam68 influence AR-V7 by altering splicing patterns [14]. Understanding these mechanisms is crucial for developing new therapies against AR-V7-mediated drug resistance.

Galectin-1 (Gal-1), a galectin family member, influences various cellular processes and is linked to cancer proliferation, splicing, and metastasis [15,16,17,18,19,20,21]. Its overexpression is associated with drug resistance mechanisms in several cancers [22]. For example, Gal-1 induced autophagy in lung adenocarcinoma and HCC enhances resistance to cisplatin [23]. In glioblastoma, Gal-1 is upregulated by temozolomide, contributing to resistance [24]. In addition, in hepatocellular carcinoma, Gal-1 promotes resistance to sorafenib by activating the FAK/PI3K/AKT pathway [25]. These roles highlight Gal-1 as a critical target for overcoming drug resistance and improving cancer treatment outcomes.

We previously showed that elevated Gal-1 is associated in prostate cancer progression and tumorigenesis [26]. However, although Gal-1 plays crucial roles in PCa, the role of Gal-1 in resistance to enzalutamide is still unexamined. In this study, we found that Gal-1 is upregulated in enzalutamide resistance cell lines but not in enzalutamide -sensitive cell lines. Treatment with enzalutamide induced Gal-1 expression in Gal-1 null C4-2B cells, promoting treatment resistance. Knocking down Gal-1 improved the response to enzalutamide treatment. Furthermore, we demonstrated that Gal-1 plays a critical role in regulating the expression of both AR full length and AR-V7. Importantly, our study showed that the Gal-1 inhibitor LLS30 significantly enhances the antitumor efficacy of enzalutamide both in vitro and in vivo. These findings elucidate a potential mechanism of resistance to enzalutamide and propose a strategy to enhance therapy outcomes for enzalutamide resistant PCa.

## 2. Material and Methods

### 2.1. Cell Lines

The cell lines used in this study include human CWR-R1, 22RV1, C4-2B and LNCaP cells. These cells were cultured in RPMI1640 supplemented with 10% fetal bovine serum and 1% penicillin/streptomycin, and maintained in a 5% CO_2_ atmosphere at 37 °C. Immunoblotting was employed to assess Gal-1 expression following transfection with siGal-1 or shGal-1. Routine monthly testing for Mycoplasma contamination was conducted.

### 2.2. Establishment of Gal-1 Knockdown in CRPC Cells

C4-2B ENZ-R and 22RV1 cells were seeded in 6-well plates and transfected with 50 nM of either a negative control mimic or siRNAs targeting Gal-1 (Qiagen, Hilden, Germany), including siGal-1#1 (5′-AAGCTGCCAGATGGATACGAA-3′) and siGal-1#2 (5′-CGCCAACACCATCGTGTGCAA-3′), using Lipofectamine 2000 (Life Technologies, Carlsbad, CA, USA) according to the manufacturer’s instructions. C4-2B ENZ-R cells were infected with either control or Gal-1-specific shRNA lentiviral particles (sc-35441-v, Santa Cruz Biotechnology, Dallas, TX, USA) at a multiplicity of infection (MOI) of 10 for 24 h, using 8 mg/mL polybrene to enhance infection efficiency. Gal-1 knockdown was validated through immunoblotting.

### 2.3. Cell Viability and Apoptosis Assay

To evaluate cell viability, 5 × 10^3^ cells were plated per well in 96-well plates and left to adhere for overnight, prior to being treated with drugs for 72 h. After this initial incubation, the medium was replaced, and cells were exposed to designated concentrations of LLS30 or enzalutamide. Stock solutions of LLS30 and enzalutamide (10 mM) were first prepared in 100% DMSO (100× concentration). A working solution of LLS30 at 100 µM in 1% DMSO was prepared by diluting the stock solution 1:100 with cell culture medium, followed by successive two-fold serial dilutions. Cell viability was assessed after 72 h of incubation with drugs using the MTT assay. For apoptosis evaluation, caspase-3/7 activity was measured after treating cells with 25 μM enzalutamide, or 3 μM LLS30 for 24 h, using a luminescent caspase-Glo 3/7 assay kit from Promega (Madison, WI, USA).

### 2.4. Colony Formation

500 cells were seeded in 6-well plates in triplicate. After 24 h, the cells were treated with 0.03% DMSO or LLS30 at concentrations of 1.5 µM or 3 µM. The medium, containing either DMSO or the specified concentrations of LLS30, was refreshed every two days. After a period of 10 days, the cells were fixed with 100% methanol for 5 min at room temperature and subsequently stained with a 0.5% crystal violet solution in 20% methanol for 30 min at room temperature. Colonies were then quantified using ImageJ (version 1.54).

### 2.5. Selection of Enzalutamide Resistant Cell Lines

C4-2B cells were initially exposed to 10 µM enzalutamide for 72 h. The surviving cells were then re-seeded and given a week to recover. Over the next four months, enzalutamide-resistant cells were developed by gradually increasing the concentrations of enzalutamide from 10 to 50 µM. In parallel, age-matched parental cells that did not undergo treatment were also maintained. The developed resistant cells were continuously cultured in 20 µM enzalutamide.

### 2.6. Immunoblotting Analysis

The immunoblot assay was performed using the protocol previously described [27]. In summary, cells were lysed with RIPA buffer and kept on ice for 20 min. After centrifugation, the total cell lysates were collected, and protein concentrations were determined using the BCA assay. Samples containing 20 μg of protein were denatured in 2X Laemmli SDS-PAGE sample buffer, resolved on 12% SDS-PAGE gels, and then transferred onto PVDF membranes. The membranes were blocked using 10% non-fat dried milk in Tris-buffered saline and subsequently incubated overnight at 4 °C with specific primary antibodies targeting Gal-1 (Abcam, Cambridge, UK), AR (Cell Signaling, Danvers, MA, USA), AR-V7 (Precision Antibody, Columbia, MD, USA), or beta-actin (Cell Signaling, Danvers, MA, USA). After three washes with TBS-T, the membranes were treated with HRP-linked secondary antibodies at 37 °C for one hour. The chemiluminescence signal was visualized using ECL substrate and captured with a CCD camera.

### 2.7. Construction of AR and AR-V7 Reporter Plasmids and Reporter Assays

The PSA promoter was subcloned as a KpnI-XhoI fragment into the pGL3-basic luciferase expression vector (Promega, Madison, WI, USA) to generate the plasmid pGL3-PSA. Genomic DNA from C4-2B cells was used as the template to amplify the PSA promoter region using the primers 5′-AATTGGTACCCATTGTTTGCTGCACGTTGGAT-3′ and 5′-AATTCTCGAGTCCGGGTGCAGGTGGTAAGCTTGG-3′ [28]. For the AR-V7 reporter plasmid, the promoter fragment of the AR-V7-specific target gene UBE2C was cloned into the pGL3-Basic vector. Total DNA from 22Rv1 cells served as the template to amplify the UBE2C promoter region using the primers 5′-AATTGGTACCTGTTCCCACGCGGAGTAAG-3′ and 5′-AATTCTCGAGGGGGGTGGTCCTAGAAATC-3′. The PCR products were then inserted into the pGL3-Basic at the KpnI–XhoI cloning site to generate pGL3-UBE2C [29]. To assess the impact of Gal-1 protein and investigate the influence of drugs on AR and AR-V7 transcription activity, 1 × 10^5^ cells were seeded in a 24-well plate and transiently cotransfected with 0.5 μg of pGL3-PSA and either 50 nM siRNA control or siRNA targeting Gal-1, along with 50 ng pRL-TK (to assess AR signaling) or pGL3-UBE2C and pRL-TK (to assess AR-V7 signaling) using Lipofectamine 2000 (Invitrogen, Waltham, MA, USA). In a separate experiment to determine the impact of drugs on AR and AR-V7 transcription activity, cells were transfected with the same constructs (pGL3-PSA and pRL-TK for AR signaling or pGL3-UBE2C and pRL-TK for AR-V7 signaling). After 24 h, these cells were treated with enzalutamide (25 µM) or LLS30 (5 µM). Luciferase activity was measured 72 h after transfection in both experiments using the dual-luciferase reporter assay system as per the manufacturer’s instructions (Promega, Madison, WI, USA). The firefly luciferase enzyme activity was normalized to the Renilla luciferase enzyme activity. The experimental samples were then normalized to control samples, resulting in values for the fold reduction of firefly luciferase activity.

### 2.8. In Vivo Animal Assays

Animal experiments in this study were approved by the Institutional Animal Care and Use Committee (IACUC) of the University of California, Davis (D16-00272). A total of 1 × 10^6^ C4-2B parental or C4-2B ENZ-R cells were suspended in 0.2 mL of matrigel (BD Biosciences, San Jose, CA, USA) and subcutaneously inoculated into the flanks of male nude mice (Jackson Laboratory, Bar Harbor, ME, USA). Once tumor volumes reached approximately 100 mm^3^, the mice were treated with 25 mg/kg enzalutamide via oral gavage (o.p.) for two weeks. For the prostate cancer orthotopic xenograft model, 1 × 10^6^ cells in 50 µL of a 1:1 mixture of culture medium and matrigel were injected into the prostate gland of each mouse. The mice were divided into four groups: (1) shControl transfected cells + vehicle (0.5% Tween 80/PBS) administered via o.p., (2) shGal-1 transfected cells + vehicle (0.5% Tween 80/PBS, o.p.), (3) shControl transfected cells + enzalutamide (25 mg/kg in 0.5% Tween 80/PBS, o.p.), and (4) shGal-1 transfected cells + enzalutamide (25 mg/kg in 0.5% Tween 80/PBS, o.p.). Two weeks after treatment, bioluminescence signals were detected by the IVIS 200 Imaging System (version 2.50) (Caliper LifeSciences, Hopkinton, MA, USA), five minutes after intraperitoneal injection of 100 mg/kg d-luciferin. Bioluminescence signals were quantified with Aura software (version is 1.07.84_V2). For subcutaneous xenografts models, 1× 10^6^ C4-2B ENZ-R cells were suspended in 0.2 mL of matrigel and subcutaneously inoculated into the flanks of male nude mice. Once the tumor volumes reached approximately 100 mm^3^, the mice were randomly allocated into four groups (*n* = 6) and underwent daily treatment for two weeks. Treatments administered included a vehicle (8.7% alcohol/8.7% Tween 80/PBS) via intraperitoneal (i.p.) injection, enzalutamide (25 mg/kg in 0.5% Tween 80/PBS) via o.p., LLS30 (10 mg/kg in 8.7% alcohol/8.7% Tween 80/PBS) via i.p. injection, and a combination treatment of enzalutamide (25 mg/kg, o.p.) and LLS30 (10 mg/kg, i.p.). Tumor volumes were consistently monitored throughout the experiment. At week 7, the mice were sacrificed, and tumors were excised for immunohistochemical staining of Ki-67 and cleaved caspase-3.

### 2.9. Immunohistochemical Staining

Paraffin embedded slides were dewaxed with two xylene washes, followed by rehydration steps using 100% ethanol for 5 min, 95% ethanol for 5 min, and 80% ethanol for 5 min, consecutively, and then rinsed in PBS. Antigen retrieval was carried out using a sodium citrate buffer (10 mmol/L, pH 6.0) heated to 95 °C to 100 °C for 20 min. Once cooled to room temperature, the sections were washed with PBS and treated with 1% H_2_O_2_ to block endogenous peroxidase activity and Power Block (BioGenex, Fremont, CA, USA) to prevent nonspecific binding, each for 5 min at room temperature. Sections were then incubated overnight with specific primary antibodies against cleaved caspase-3 or Ki-67 (both from Cell Signaling Technology, Danvers, MA, USA). Following another PBS rinse, sections were incubated with a biotin-conjugated goat anti-rabbit IgG (BioGenex, Fremont, CA, USA) as the secondary antibody. This was followed by a 20-min incubation with streptavidin-conjugated horseradish peroxidase (HRP; BioGenex, Fremont, CA, USA) at room temperature. HRP activity was visualized using diaminobenzidine tetrahydrochloride (DAB) as the substrate (BioGenex, Fremont, CA, USA). Finally, nuclei were counterstained with hematoxylin (Cell Signaling Technology, Danvers, MA, USA).

### 2.10. Transcriptome Sequencing

RNA-seq data analysis involved extracting total RNA from both control and LLS30-treated C4-2B ENZ-R cells, utilizing the PureLink RNA Mini Kit (Invitrogen, Waltham, MA, USA) according to the manufacturer’s guidelines. RNA quality was assessed with the Agilent 2100 Bioanalyzer system (Agilent Technologies, Santa Clara, CA, USA). Subsequently, the mRNA sequencing library was prepared, and paired-end sequencing was performed on the Illumina HiSeq 4000 Sequencing System. Gene color maps, utilizing a list of AR-related gene symbols, were created on the MORPHEUS website (https://software.broadinstitute.org/morpheus/ accessed on 30 November 2024) [28].

### 2.11. Statistical Analysis

In vitro experiments were conducted in triplicate across two independent experiments, and the results are presented as the mean ± SD. The student’s *t*-test (two-tailed) was employed for comparing datasets between two groups with similar variance. *p* value < 0.05 was considered indicative of a statistically significant difference. Statistical differences, when compared with controls, are denoted as * (*p* < 0.05), ** (*p* < 0.01), or *** (*p* < 0.001).

### 2.12. Data Sharing Statement

We are dedicated to providing materials and data to legitimate researchers upon reasonable request, in accordance with the Material Transfer Agreement (MTA).

## 3. Results

### 3.1. Gal-1 Overexpression Is Associated with Enzalutamide Resistance

**Figure 1:**
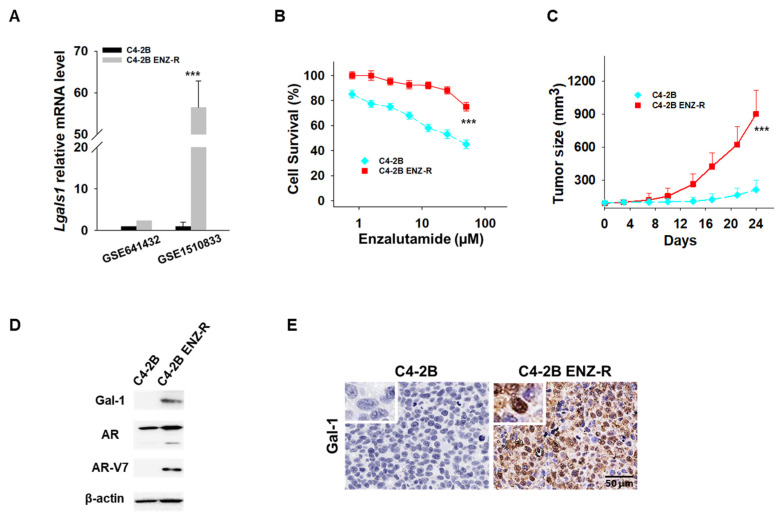
Gal-1 expression in enzalutamide-resistant cells. (**A**) Lgals1 transcript levels from two GEO datasets [30,31]. (**B**) In vitro antiproliferative activity of enzalutamide in parental C4-2B and C4-2B ENZ-R cells. Cell viability was assessed using the MTT assay after 72 h of drug incubation. (**C**) Tumor growth curves (*n* = 6 mice per group) showing the effect of enzalutamide (25 mg/kg) on the growth of parental C4-2B and C4-2B ENZ-R tumor xenografts in vivo. (**D**) Immunoblots demonstrating endogenous Gal-1 expression in parental C4-2B and C4-2B ENZ-R cells. (**E**) Representative images of Gal-1 immunohistochemistry in parental C4-2B and C4-2B ENZ-R tumor xenografts. *** *p* < 0.001; 2-tailed Student’s *t*-test. Data shown are mean ± SD. The uncropped bolts are shown in Supplementary Materials.

We have previously demonstrated the absence of Gal-1 expression in C42B enzalutamide-sensitive cells and high expression of Gal-1 in 22RV1 enzalutamide-resistant cells [26]. This differential expression suggests Gal-1’s involvement in the resistance mechanism to enzalutamide in PCa cells. However, the expression level of Gal-1 in enzalutamide-resistant PCa has not been examined. In our initial analysis, we examined published gene expression datasets (GEO: GSE641432 and GSE1510833) containing C4-2B and C4-2B enzalutamide-resistant (C4-2B ENZ-R) cells [30,31]. We found that Gal-1 was significantly upregulated in the C4-2B ENZ-R cells compared to the parental lines, with fold changes of 2.4 in dataset GSE641432 and 56.5 in dataset GSE1510833 (Figure 1A). To confirm that the increased expression of Gal-1 is associated with enzalutamide treatment, we established a C4-2B ENZ-R cell line by subjecting Gal-1 null C4-2B cells to chronic treatment with 10–50 µM enzalutamide for over four months. The acquired resistance of C4-2B ENZ-R cells to enzalutamide was verified. As shown in Figure 1B,C, cell growth assays and in vivo mouse studies indicated that C4-2B ENZ-R cells and xenografts showed a reduced response to enzalutamide, with minimal effects observed compared to C4-2B parental cells. Importantly, these resistant cells showed increased expression of Gal-1 both in vitro and in vivo (Figure 1D,E). These findings suggested that elevated Gal-1 expression may contribute to resistance to enzalutamide.

### 3.2. Knockdown of Gal-1 Overcomes Resistance and Enhances the Efficacy of Enzalutamide in Enzalutamide-Resistant PCa Cells

**Figure 2:**
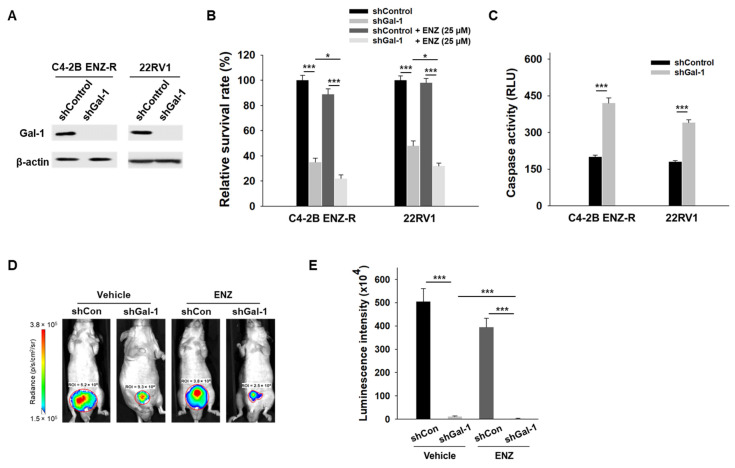
Effect of Gal-1 knockdown in enzalutamide-resistant PCa cells. (**A**) Immunoblotting of Gal-1 and β-actin in C4-2B ENZ-R and 22RV1 cells were infected with either control or Gal-1-specific shRNA lentiviral particles. (**B**) Enzalutamide-resistant C4-2B ENZ-R and 22RV1 cells were transfected with control shRNA or shRNA against Gal-1, and then treated with 25 µM enzalutamide. Cell survival was determined by MTT. (**C**) Caspase-3/7 activities in C4-2B ENZ-R and 22RV1 72 h after transfection with shRNA. (**D**) Bioluminescent images show the growth of orthotopic implanted luciferase-tagged C4-2B ENZ-R cells (stably transfected with shRNA or Gal-1 shRNA) in nude mice after 2 weeks (*n* = 4 mice per group). (**E**) Quantification of the tumor bioluminescent signal. * *p* < 0.05, *** *p* < 0.001; 2-tailed Student’s *t*-test. Data shown are mean ± SD. The uncropped bolts are shown in Supplementary Materials.

We further investigated whether Gal-1 knockdown could suppress the growth of enzalutamide-resistant cells during enzalutamide treatment. Gal-1 knockdown was achieved using shRNA, which induces stable, long-term gene silencing, making it suitable for in vivo assays (Figure 2A). As shown in Figure 2B, Gal-1 knockdown significantly reduced the growth of both C4-2B ENZ-R and 22RV1 cells. Notably, the combination of Gal-1 knockdown and enzalutamide treatment significantly reduced the viability of both cell lines. In addition, Gal-1 knockdown induced apoptosis in both C4-2B ENZ-R and 22RV1 cells (Figure 2C). We extended our studies in vivo to determine if Gal-1 knockdown could inhibit the growth of enzalutamide-resistant cells, both with and without enzalutamide treatment. C4-2B ENZ-R cells expressing control shRNA or shGal-1 were orthotopically injected into castrated nude mice. Consistent with the in vitro results, in vivo tumorigenesis studies demonstrated that Gal-1 knockdown significantly slowed tumor growth, particularly when combined with enzalutamide treatment (shGal-1 combined with enzalutamide vs. shGal-1 alone, *p* < 0.001; the mean luminescence intensity was 2.3 × 10^4^ for the group treated with shGal-1 combined with enzalutamide compared with 11.5 × 10^4^ for the shGal-1 transfected group) (Figure 2D,E). In contrast, enzalutamide treatment alone had minimal impact on the growth of control shRNA-transfected cells. These results collectively confirm that inhibiting Gal-1 expression could inhibit the growth of enzalutamide-resistant cells and sensitize them to enzalutamide treatment.

### 3.3. Gal-1 Knockdown Abrogates Expression of AR FL and AR-V7

**Figure 3:**
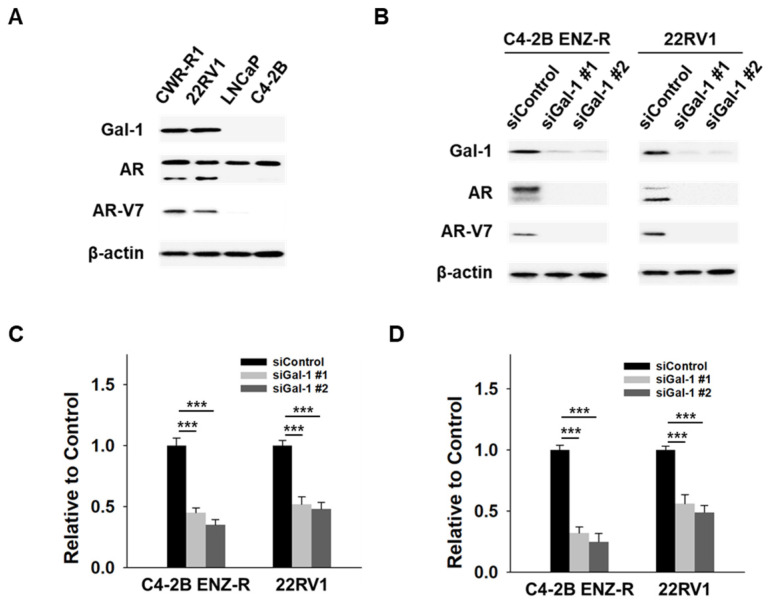
Effect of Gal-1 knockdown on AR and AR-V7 pathways in enzalutamide-resistant PCa cells. (**A**) Immunoblots demonstrating the endogenous expression of Gal-1, AR, AR-V7 and β-actin in CWR-R1 and 22RV1 (enzalutamide-resistant cells), as well as in LNCaP and C4-2B (enzalutamide-sensitive cells). (**B**) Immunoblotting of Gal-1, AR, AR-V7 and β-actin in C4-2B ENZ-R and 22RV1 cells transfected with 40 nM control siRNA or siGal-1 for 72 h. (**C**) Reporter gene assays assessing the transcriptional activity of AR and (**D**) AR-V7. C4-2B ENZ-R and 22RV1 cells co-transfected with 40 nM control siRNA or siRNA targeting Gal-1 with 0.5 μg of PSA promoter expression plasmids (pGL3-PSA) or UBE2C promoter expression plasmids (pGL3-UBE2C), and 50 ng TK promoter-driven Renilla luciferase reference plasmid (pRL/TK). Luciferase activity was measured 72 h post-transfection using the dual-luciferase reporter assay system. *** *p* < 0.001; 2-tailed Student’s *t*-test. Data shown are mean ± SD. The uncropped bolts are shown in Supplementary Materials.

Sustained AR signaling and the presence of AR-V7 are key mechanisms of enzalutamide resistance [7,8,9]. In our studies, we observed that knocking down Gal-1 suppressed the growth of enzalutamide -resistant cells, suggesting a potential regulatory role for Gal-1 in the expression of both full-length AR (AR-FL) and AR-V7. To further investigate this hypothesis, we conducted siRNA-mediated knockdown experiments targeting Gal-1 in enzalutamide-resistant cells and subsequently measured the expression levels of AR-FL and AR-V7. Both C4-2B ENZ-R and 22RV1 cells exhibited elevated levels of AR-FL and AR-V7 protein (Figure 3A). Notably, silencing Gal-1 significantly decreased the expression levels of both AR-FL and AR-V7 in these cell lines (Figure 3B). Given that AR and AR-V7 exert their functions primarily through transcriptional activity, we utilized reporter assays to evaluate the impact of Gal-1 knockdown on the transcriptional activity of AR and AR-V7. Dual reporter assays demonstrated that Gal-1 knockdown significantly reduced the luciferase activity of the promoter luciferase reporter plasmid pGL3-PSA and pGL3-UBE2C in both C4-2B ENZ-R and 22RV1 cells (Figure 3C,D). This indicates that Gal-1 knockdown diminished the transcriptional activities of AR and AR-V7. These findings indicate that Gal-1 contributes to the transcriptional regulation of AR and AR-V7, and that its inhibition leads to decreased AR signaling and cell proliferation.

### 3.4. Effects of LLS30 on AR-FL and AR-V7 Signaling Are Associated with Its Effect on Gal-1 Knockdown

**Figure 4:**
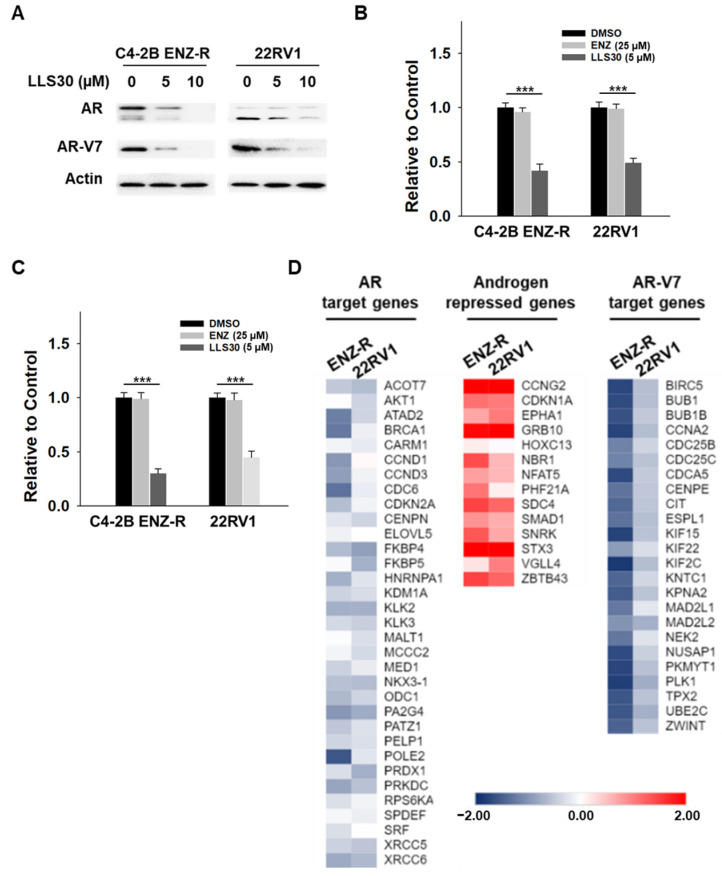
Effect of LLS30 on AR and AR-V7 pathways in enzalutamide-resistant PCa cells. (**A**) Immunoblotting of AR, AR-V7, and β-actin in C4-2B ENZ-R and 22RV1 cells treated with 5 µM or 10 µM LLS30. (**B**) Reporter gene assays assessing the transcriptional activity of AR and (**C**) AR-V7. C4-2B ENZ-R and 22RV1 cells were co-transfected with 0.5 μg of PSA promoter expression plasmid (pGL3-PSA) or UBE2C promoter expression plasmid (pGL3-UBE2C), and 50 ng pRL/TK. At 24 h post-transfection, cells were treated with 25 µM enzalutamide or 5 µM LLS30. Following 48 h of treatment, luciferase activity was measured using the dual-luciferase reporter assay system. (**D**) LLS30 regulated the AR-FL and AR-V7 gene programs. The heat map displays fold changes in gene expression, as detected by RNA-seq in C4-2B ENZ-R and 22RV1 cells treated with 5 μM LLS30 for 24 h, compared to control (DMSO). Left: AR target genes. Middle: androgen-repressed genes. Right: AR-V7 target genes. *** *p* < 0.001. The uncropped bolts are shown in Supplementary Materials.

To target Gal-1, we developed LLS30, an allosteric inhibitor that reduces Gal-1’s binding affinity to its partners and consequently inhibits its functions [26]. Here, we tested whether LLS30 has similar effects on AR-FL and AR-V7 expression, as observed with Gal-1 knockdown. As shown in Figure 4A, LLS30 inhibited the expression of AR-FL and AR-V7 in a dose-dependent manner. We further evaluated the effects of LLS30 on the transcriptional activity of AR and AR-V7. While enzalutamide treatment did not affect the promoter activity of AR and AR-V7 (Figure 4B,C), LLS30 significantly suppressed the transcriptional activity of AR and AR-V7 in both C4-2B ENZ-R and 22RV1 cells (Figure 4B,C). In addition, RNA-Seq experiments were conducted to examine whether LLS30 affected AR or AR-V7-dependent endogenous gene expression on C4-2B ENZ-R and 22RV1 cells. The data showed that LLS30 inhibited the expression of AR target genes and androgen-induced genes that promote prostate cancer proliferation (Figure 4D). In contrast, androgen-repressed genes that inhibit prostate cancer proliferation, such as CCNG2 and CDKN1A, were induced by LLS30 (Figure 4D). Furthermore, LLS30 also inhibited the expression of AR-V7-specific target genes (Figure 4D). These results collectively indicate that LLS30 regulates AR-FL and AR-V7 transcriptional activity, consistent with the effects observed with Gal-1 knockdown. This suggests that LLS30 suppresses AR-FL and AR-V7 functions, likely through the inhibition of Gal-1.

### 3.5. Synergistic Effects of LL30 and Enzalutamide on Enzalutamide-Resistant CRPC Cells

**Figure 5:**
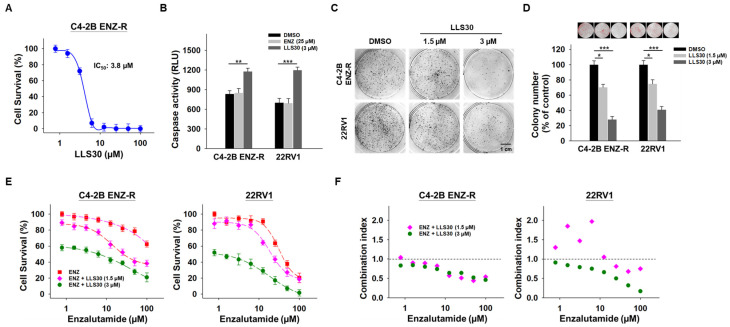
Effect of LLS30 in enzalutamide-resistant cells in vitro. (**A**) Cell survival of C4-2B ENZ-R cells treated with indicated concentrations of LLS30 for 72 h. IC_50_ of LLS30 on C4-2B ENZ-R is 3.8 μM. (**B**) Caspase-3/7 activities in C4-2B ENZ-R and 22RV1 cells after 24 h treated with DMSO or 25 μM enzalutamide or 3 μM LLS30. (**C**) Effect of LLS30 on colony formation in enzalutamide-resistant cells. Crystal violet staining was performed on colonies derived from an equal number of cells treated with 1.5 or 3 μM LLS30. (**D**) Quantification of crystal violet-stained colonies. Representative images showed colony analysis by ImageJ (upper panel) and the average relative ratio of crystal violet-stained colonies (lower panel). Colony numbers were assessed 10 days post-seeding. (**E**) Combination activity for enzalutamide and LLS30 for 72 h. (**F**) Combination index (CI) values for enzalutamide and LLS30 in C4-2B ENZ-R and 22RV1 under various combination treatments. CI < 1 indicated synergistic effects represented in green color. * *p* < 0.05, ** *p* < 0.01, *** *p* < 0.001; 2-tailed Student’s *t*-test. Data shown are mean ± SD.

Considering the pivotal role of Gal-1 in enzalutamide-resistant PCa cells, we investigated whether LLS30 exhibits any inhibitory effects on the growth of these cells. LLS30 was found to be effective against C4-2B ENZ-R cells, with an IC_50_ value of 3.8 µM (Figure 5A). In addition, LLS30 induced apoptosis (Figure 5B) and suppressed colony formation in C4-2B ENZ-R cells (Figure 5C,D). Given that LLS30 can suppress AR-V7 expression, we examined whether it could re-sensitize enzalutamide-resistant cells to enzalutamide. We treated C4-2B ENZ-R cells with LLS30 and enzalutamide for 72 h and analyzed cell survival. As expected, enzalutamide alone did not significantly impact the growth rate of CRPC C4-2B and 22RV1 cells (Figure 5E). However, when treated with a combination of enzalutamide and LLS30, the growth rate of both C4-2B ENZ-R and 22RV1 cells was significantly suppressed (Figure 5D). Moreover, we conducted a synergistic analysis to determine whether LLS30 worked synergistically with enzalutamide. Importantly, the results revealed that the combination of LLS30 and enzalutamide synergistically induced cell death, as evidenced by a combination index of less than 1 for most drug combination concentrations tested (Figure 5F). These findings suggest that LLS30 not only inhibited the growth of enzalutamide-resistant PCa cells but also enhanced the efficacy of enzalutamide treatment.

### 3.6. LLS30 Restores Responsiveness of Enzalutamide-Resistant Cells to Enzalutamide Treatment In Vivo

**Figure 6:**
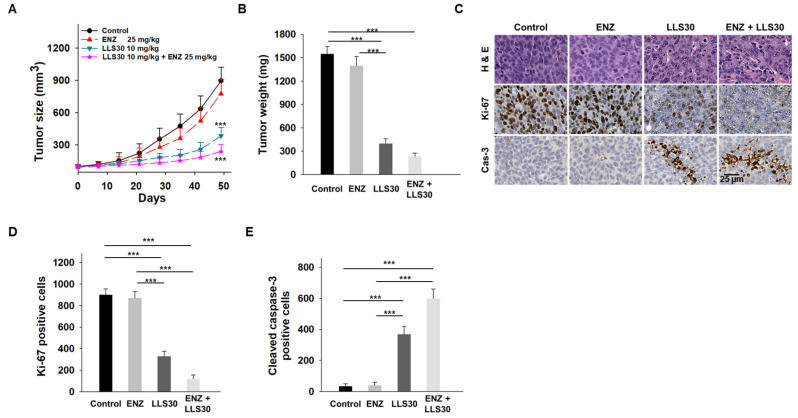
Effects of LLS30 in enzalutamide-resistant PCa cells in vivo. (**A**) Tumor growth curves (*n* = 6 mice per group) illustrating the effects of enzalutamide, LLS30, and their combination on the growth of C4-2B ENZ-R tumor xenografts in vivo. (**B**) Tumor xenograft weights. (**C**) Representative images of H&E, Ki-67, and cleaved caspase-3 immunohistochemistry in C4-2B ENZ-R tumor xenografts. (**D**) Quantification of Ki-67 and (**E**) cleaved caspase-3 immunostaining. Positive cells were counted in three randomly chosen areas. *** *p* < 0.001; 2-tailed Student’s *t*-test. Data shown are mean ± SD.

We next evaluated the effectiveness of LLS30 treatment, both alone and in combination with enzalutamide, in castrated nude mice bearing C4-2B ENZ-R xenografts. Intraperitoneal administration of LLS30 at a dose of 10 mg/kg significantly suppressed the growth and tumor weight of C4-2B ENZ-R xenografts (Figure 6A,B). The enzalutamide treatment alone, administered via oral gavage, had a minimal effect on the growth of these xenografts. Importantly, the combination of LLS30 and enzalutamide resulted in a significant reduction in tumor volume compared to the group treated with enzalutamide alone. To further understand the impact on tumor proliferation and apoptosis, excised tumors were analyzed for cleaved caspase-3 and Ki-67 levels. In LLS30-treated tumors, there was an 11-fold increase in cleaved caspase-3 positive cells and a significant 3-fold decrease in Ki-67 positive cells compared to the control group (Figure 6C–E). Notably, the combination of LLS30 and enzalutamide treatment resulted in a dramatic increase in cleaved caspase-3 positive cells and a marked decrease in Ki-67 positive cells (Figure 6C–E). These findings suggest that LLS30 may restore the responsiveness of enzalutamide-resistant cells to enzalutamide treatment, highlighting its potential as a therapeutic agent in overcoming resistance to enzalutamide in CRPC.

## 4. Discussion

Enzalutamide is commonly used for advanced prostate cancer, particularly CRPC, but often encounters challenges like drug resistance, largely due to mutations in the AR, increased expression of splice variants like AR-V7, and activation of alternative signaling pathways [5,32,33,34]. Cross-resistance with other AR-targeted therapies such as abiraterone can limit future treatment options after enzalutamide failure [9,35]. Our study discovered that enzalutamide treatment induces Gal-1 expression in C4-2B cells, contributing further to resistance. Suppressing Gal-1 not only significantly reduced AR and AR-V7 expression but also enhanced enzalutamide’s antitumor efficacy in vitro and in vivo. These findings suggest that targeting Gal-1 could improve enzalutamide therapy and offer a new approach for treating enzalutamide-resistant CRPC.

In addition to restoring enzalutamide sensitivity, our findings show that Gal-1 inhibition exerts significant anti-tumor effects independent of drug resistance, suggesting that Gal-1’s role in prostate cancer extends beyond modulating drug resistance. Indeed, our previous study demonstrated that inhibiting Gal-1 expression or function suppressed tumor growth through the inhibition of Akt signaling and AR expression. Moreover, Gal-1 inhibition reduced tumor growth in both AR-positive and AR-negative xenograft models, indicating that its impact goes beyond AR signaling. Furthermore, Gal-1 inhibition lowered metastatic potential, likely through modulation of integrin and FAK signaling, which are crucial for tumor invasion. These findings underscore Gal-1 as a promising therapeutic target for addressing prostate cancer progression beyond drug resistance, enhancing the potential of inhibitors like LLS30 in treating advanced prostate cancer.

The potential of Gal-1 as a therapeutic target is further underscored by recent studies, which have highlighted its role in mediating resistance to various anticancer therapies [22]. For instance, Gal-1 overexpression has been linked to increased levels of P-glycoprotein, a key player in drug resistance, in breast cancer cells [36]. Reducing Gal-1 levels led to decreased P-glycoprotein and increased sensitivity to paclitaxel and doxorubicin [37]. In addition, Gal-1 has been implicated in the modulation of survival pathways, helping cancer cells evade drug-induced toxicity. In HCCs, Gal-1 mediates resistance to cisplatin by inducing autophagy, thereby preventing mitochondrial potential loss and apoptosis [23]. Similarly, in neuroblastoma cells, knocking down Gal-1 increases cisplatin sensitivity by inhibiting autophagy [38]. This emerging evidence underscores the need for further research into Gal-1 inhibitors as a strategy to combat drug resistance in cancer, expanding the implications of our findings and indicating a crucial area for future studies.

Recent studies indicated that the splicing factors (e.g., U2AF65, SRSF1 and hnRNP1) is frequently aberrantly regulated in CRPC leading to increases in AR and generation of AR-V7 [14,39,40]. Concurrently, intracellular Gal-1 has been reported as splicing factor to interact with Gemin4 for the splicing of pre-mRNA [17,18]. In addition, Zhang et al. demonstrated that Gal-1 binds to the mRNAs of angiogenesis-related genes like VEGFA, EGR1, and LAMA5, suggesting that Gal-1 may influence angiogenesis through its mRNA-binding capabilities [41]. Similarly, Fang et al. revealed that Gal-1 regulates NSCLC progression through alternative splicing (AS) events. Gal-1-associated AS genes were primarily enriched in apoptosis and ErbB signaling pathways, and silencing Gal-1 led to a reduced alternative splicing ratio of BCAP29 while increasing levels of CSNKIE and MDFIC [42]. Interestingly, in this study, we observed the presence of Gal-1 accumulating in the nucleus of C4-2B ENZ-R cells (Figure 1E), which express AR-V7. This observation implies a potential involvement of nuclear Gal-1 in the process of AR alternative splicing. Further investigation is underway to determine whether Gal-1 acts as a splicing factor in the regulation of AR splicing mechanisms.

While Galectin-1 (Gal-1) has been extensively reported to regulate not only tumor-intrinsic properties but also the broader tumor microenvironment, including immunity, the experimental design of this study is primarily focused on evaluating the endogenous tumor-related effects of Gal-1. We acknowledge that the role of the immune microenvironment, particularly immune cell populations and their interactions with tumor cells, has not been explored in the current study. Future investigations will be necessary to assess the comprehensive impact of Gal-1 on immune modulation within the tumor microenvironment, providing a more holistic understanding of its role in tumor progression and immune evasion. By integrating immune profiling and functional assays in subsequent studies, we aim to elucidate the interplay between Gal-1 and tumor immunity more effectively.

Currently, no approved therapeutic compounds exist to treat patients with prostate cancer resistant to enzalutamide. However, LLS30, a novel small molecule inhibitor of Gal-1, shows promise in addressing this critical clinical challenge in enzalutamide-resistant CRPC. Our previous studies have demonstrated that LLS30 effectively treats CRPC in animal models without significant adverse effects on body weight, behavior, or serum chemistry profiles, indicating its potential as a well-tolerated therapeutic agent for CRPC treatment. Here, we demonstrated that LLS30 alone significantly suppressed the growth of C4-2B ENZ-R xenografts and enhanced the antitumor efficacy of enzalutamide. Our findings highlight synergistic treatment strategies that could effectively enhance therapy outcomes for enzalutamide resistant PCa. Despite compelling evidence implicating Gal-1 in cancer progression, there are currently no approved therapies specifically targeting Gal-1 in clinical practice. The successful development of LLS30 could revolutionize the treatment landscape for CRPC, particularly for patients who have developed resistance to enzalutamide.

## 5. Conclusions

This study highlights the significant role of Gal-1 in PCa and its contribution to drug resistance, identifying it as a potential target for innovative therapies. Notably, in patients where Gal-1 expression is induced following enzalutamide treatment, the efficacy of enzalutamide may become less effective. Given this, the integration of LLS30, a novel inhibitor of Gal-1, into treatment regimens could enhance the management of enzalutamide-resistant prostate cancer. Continued research and development of LLS30-based therapies is essential to devise effective strategies against drug resistance, addressing the specific mechanisms driving resistance in high Gal-1/AR-expressing prostate cancer, and providing a targeted, personalized approach to therapy.

## Data Availability

The raw data supporting the conclusions of this article will be made available by the authors on request.

## Supplementary Materials

The following supporting information can be downloaded at: https://www.mdpi.com/article/10.3390/cancers17030351/s1, The uncropped bolts are shown in Supplementary Materials.

## Abbreviations

PCa: Prostate Cancer, Gal-1: Galectin-1, siRNA: Small Interfering RNA, AR: Androgen Receptor, AR-FL: Androgen Receptor Full Length, AR-V7: Androgen Receptor Variant 7, CRPC: Castration-Resistant Prostate Cancer, ENZ: Enzalutamide.
