# Supplementary material for: Inhibition of Galectin-1 and Androgen Receptor Axis Enhances Enzalutamide Treatment in Enzalutamide Resistant Prostate Cancer
Source: Cancers (Basel). 2025 Jan 22;17(3):351. doi: 10.3390/cancers17030351 (PMC11816353; doi:10.3390/cancers17030351)
Supplement: Supplementary file 1 [file cancers-17-00351-s001.zip › cancers-3424353-supplementary.pptx]

## Slide 1
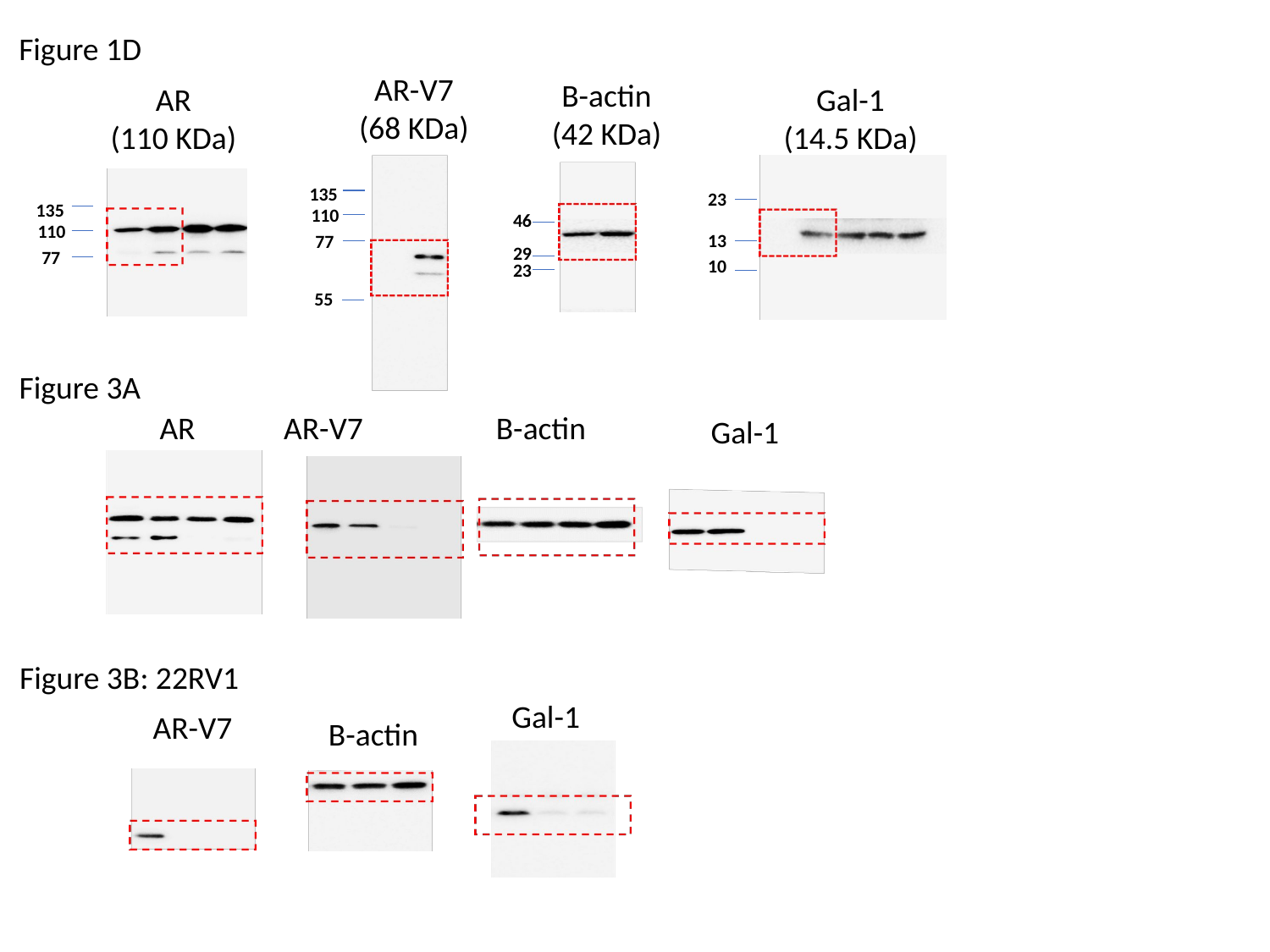

Figure 1D
AR-V7
(68 KDa)
B-actin
(42 KDa)
AR
(110 KDa)
Gal-1
(14.5 KDa)
135
23
135
110
46
110
13
77
29
77
10
23
55
Figure 3A
AR
AR-V7
B-actin
Gal-1
Figure 3B: 22RV1
Gal-1
AR-V7
B-actin

## Slide 2
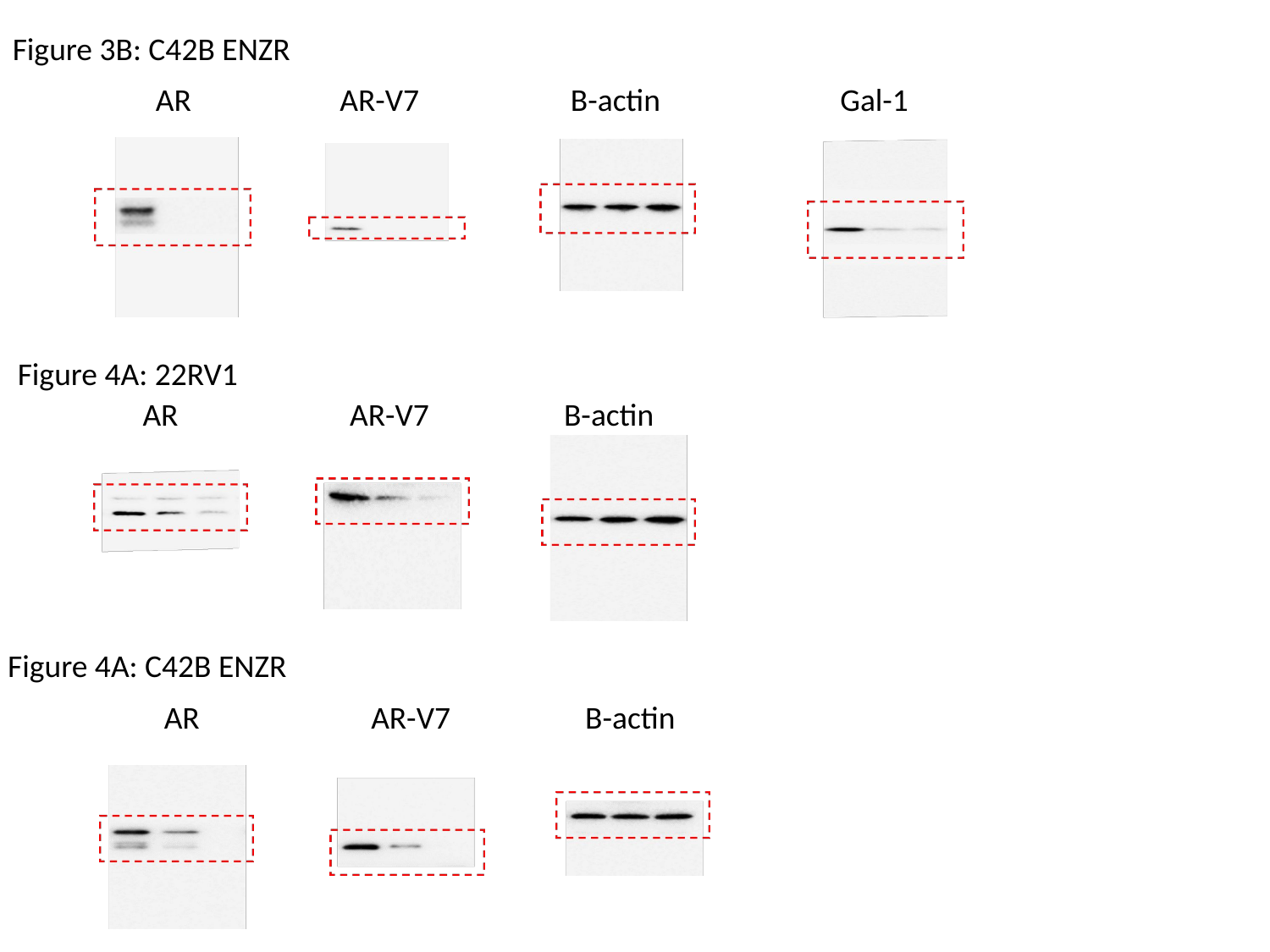

Figure 3B: C42B ENZR
AR
AR-V7
B-actin
Gal-1
Figure 4A: 22RV1
AR
AR-V7
B-actin
Figure 4A: C42B ENZR
AR
AR-V7
B-actin
