# Supplementary figures and images for: Inhibition of Galectin-1 and Androgen Receptor Axis Enhances Enzalutamide Treatment in Enzalutamide Resistant Prostate Cancer
Source: Cancers (Basel). 2025 Jan 22;17(3):351. doi: 10.3390/cancers17030351 (PMC11816353; doi:10.3390/cancers17030351)

## Slide 1
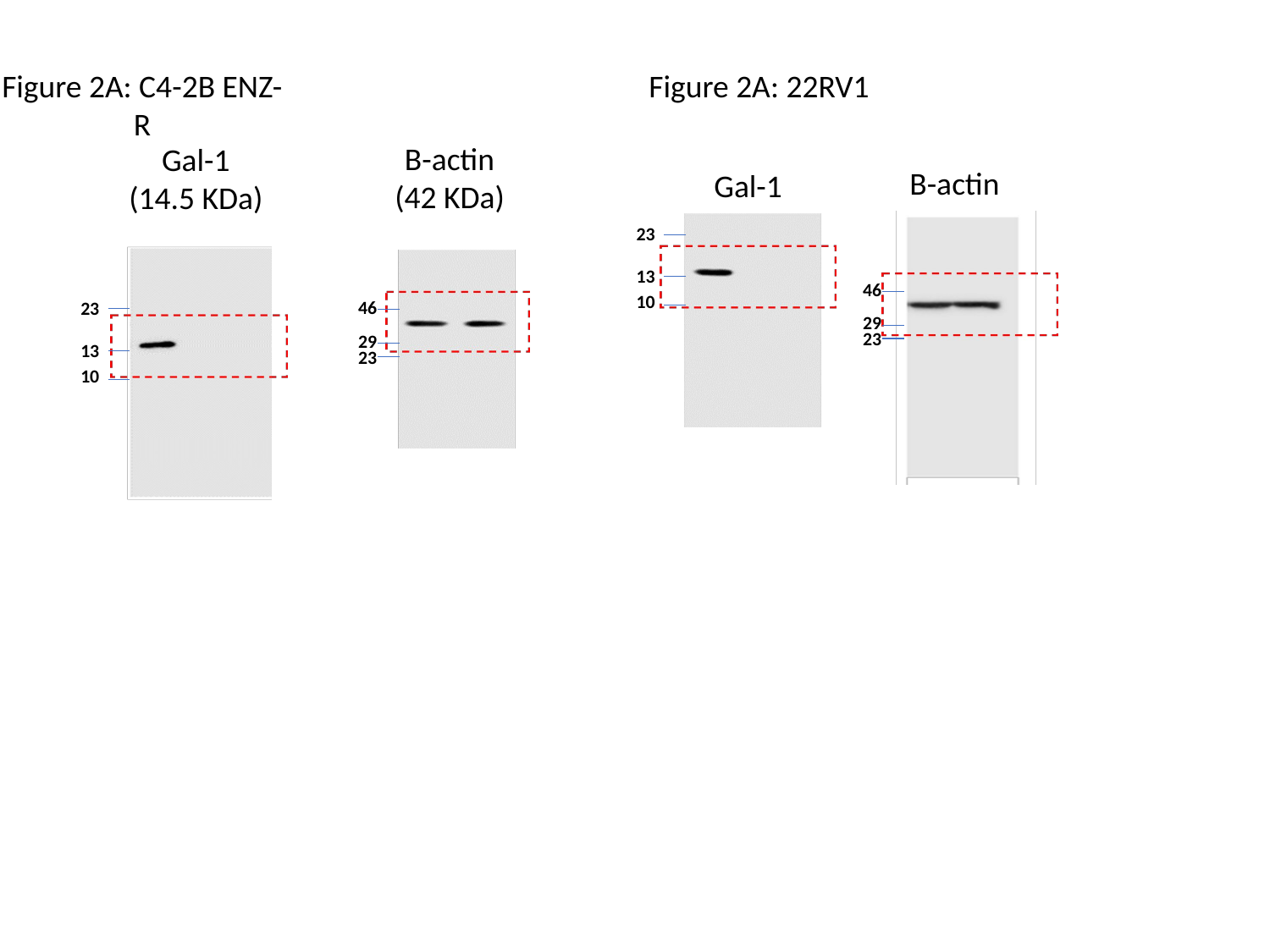

Figure 2A: C4-2B ENZ-R
Figure 2A: 22RV1
B-actin
(42 KDa)
Gal-1
(14.5 KDa)
B-actin
Gal-1
23
13
46
10
46
23
29
23
29
13
23
10

Supplement: Supplementary file 1 [file cancers-17-00351-s001.zip › Fig 2A.pptx]
